# Rising sudden cardiac deaths among young Indian actors and models: The role of steroids, extreme fitness, and societal pressure

**DOI:** 10.21542/gcsp.2025.27

**Published:** 2025-06-30

**Authors:** Shrishti P. Khetan, Shruti Suresh Suvarna

**Affiliations:** American University of Barbados, Bridgetown, Saint Michael, Barbados

## Dear Editor,

Young Indian actors and models have been experiencing sudden cardiac deaths (SCD) at an alarming rate. This growing concern is supported by recent cases involving prominent Indian TV personalities such as Sidharth Shukla, Sunjay Kapur, and Rituraj Singh. Cardiovascular disease is the leading cause of death in India, accounting for 28.1% of deaths^1^, with nearly 10.3% attributed to sudden cardiac death (SCD)^2^ in rural Southern India. SCD is disproportionately frequent among adults aged 30–50 years, including actors and models. Overall, SCD occurs 5–8 years earlier in Indians than in Western populations^2^. Globally, the incidence of SCD in young adults (<35 years) ranges from 0.47–1.21 per 100,000 person-years^3^, with higher rates among athletes due to steroid use and overtraining.

The pursuit of an idealized physique exerts significant pressure in professions such as modeling and acting, where appearance is closely tied to career success. This pressure is particularly intense in India’s entertainment industry. Research indicates that 30–40% of Indian youth (ages 18–25) internalize media-driven athletic ideals^4^, often resulting in body dissatisfaction and eating disorders. Another study^5^ also revealed that celebrities commonly report appearance anxiety and body dysmorphia due to constant scrutiny.

Although sudden cardiac death (SCD) in individuals under 40 years of age is relatively rare, it is frequently linked to undiagnosed conditions such as hypertrophic cardiomyopathy and inherited arrhythmias. However, additional modifiable factors have been recognized. Emerging evidence^6,7^ shows that extreme physical training, anabolic steroid use, and severe dietary practices can induce structural and functional heart changes, including left ventricular hypertrophy, reduced ejection fraction, plaque formation, and arrhythmias, all of which elevate SCD risk, even in those appearing outwardly healthy.

### Pathophysiology of increased cardiac risk

The intersection of extreme physical activity and steroid use presents several modifiable cardiovascular risk factors.

• **Anabolic–androgenic steroids (AAS):** Associated with myocardial hypertrophy, arrhythmias, thrombosis, and SCD.
• **Caloric restriction:** Leads to bradycardia, decreased cardiac output, and impaired myocardial function.
• **Dehydration from excessive exercise:** Causes electrolyte imbalances, hemoconcentration, and arrhythmogenic potential.
• **Overtraining syndrome:** Results in chronic sympathetic activation, elevated blood pressure, and reduced myocardial recovery.

The rising incidence of SCD among young Indian actors and models attributed to these practices highlights critical public health implications. Routine cardiovascular screening should be mandatory for individuals engaged in extreme fitness activities or anabolic steroid use. Mandatory ECG-based screening in Italy reduced SCD in athletes from **3.6 to 0.4 per 100,000 per year** over two decades^8^. Public health measures and mass media campaigns are necessary to raise awareness of the dangers associated with steroids, extreme physical training, and dietary restrictions. Studies have shown that CPR training and AED placement improve survival rates in cases of sudden cardiac arrest^9^ and are more cost-effective than widespread ECG screening. Emerging technologies, including smartwatch-based ECGs, show promise as scalable and affordable tools, with early data indicating over 90% sensitivity^10^, although validation is ongoing. Additionally, stricter regulations governing the sale and marketing of anabolic steroids and dietary supplements in India are essential to prevent such incidents.

**Figure 1.**
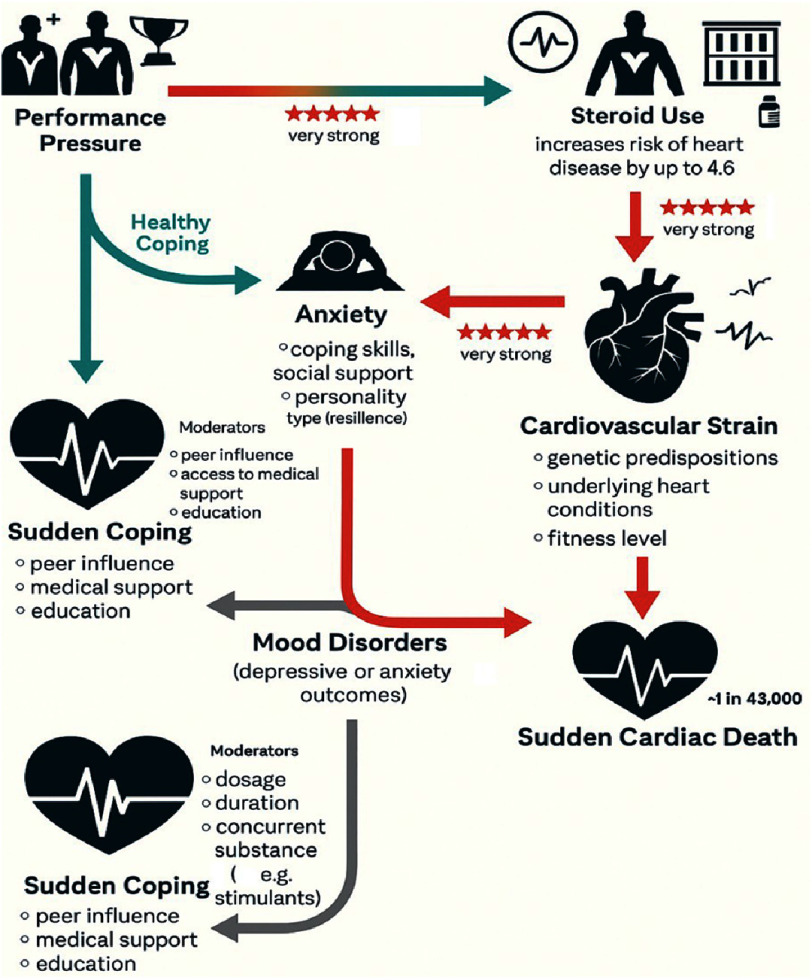
pathway to sudden cardiac death: from external pressures to fatal outcomes in the entertainment industry

Nevertheless, in India, there are significant barriers to implementation, including costs, gaps in infrastructure, false-positive rates, lack of access to trained personnel, and lack of follow-up systems. Thus, the use of targeted screening, community CPR/AED education and training, and the deployment of validated digital health interventions may offer an equitable and practical approach in the future.

It is crucial for the entertainment and fitness industries to prioritize health over aesthetics. Promoting natural fitness, balanced training regimens, and heart-healthy practices can reduce the incidence of preventable cardiac death. Healthcare professionals, policymakers, and industry leaders must collaborate to implement these interventions.

The alarming rise in SCD among young Indian actors and models is a preventable public health issue fueled by societal pressures and inadequate regulation. Through enhanced cardiovascular screening, greater regulation of supplements, and a cultural shift towards healthier body ideals, we can protect the health of young professionals and prevent further tragic losses in the future.
